# A commentary on ‘Association of postoperative outcomes with geriatric nutritional risk index after conventional and robotic-assisted total knee arthroplasty: a randomized controlled trial’

**DOI:** 10.1097/JS9.0000000000001630

**Published:** 2024-05-17

**Authors:** Chun-Ying Huang, Ching-Hua Hsieh

**Affiliations:** Department of Trauma Surgery, Kaohsiung Chang Gung Memorial Hospital, and Chang Gung University College of Medicine, Kaohsiung, Taiwan

*Dear Editor,*

We read with considerable interest the recent *International Journal of Surgery* article titled ‘Association of postoperative outcomes with geriatric nutritional risk index after conventional and robotic-assisted total knee arthroplasty: a randomized controlled trial’^1^. This study mainly found that elderly adults with a geriatric nutritional risk index (GNRI) >100 have lower hip–knee–ankle (HKA) deviation when receiving robotic-assisted total knee arthroplasty (RA-TKA) compared to those receiving conventional TKA, while the knee society score (KSS) score is similar. However, among those with a GNRI ≤100, the KSS score is higher in those receiving RA-TKA, while the HKA deviation is similar.

While acknowledging the arduous effort put forth by Dr Liu *et al*., I would like to express a few reservations pertaining to this research: Initially, the author presented that a good nutrition status is the reason for a better outcome; however, it had to be mentioned that the relationship identified in the study is an association but not cause–effect relationship, particularly since there was no comparison of patients with a GNRI >100 versus a GNRI ≤100 in the population receiving different kinds of operations. In addition, the KSS is a scoring system designed to evaluate both objective and subjective results of total and partial knee arthroplasty. It is made up of five parts: patient demographics, an objective knee assessment performed by the surgeon, patient expectations, patient satisfaction, and a functional activity score. Given that the indication for allocating individuals to receive RA-TKA or conventional TKA is uncertain, the KSS can be influenced by patient expectations and satisfaction, and the patient population is limited, the results may be heavily influenced by selection bias. For example, people undergoing RA-TKA may have superior social-economic position, a better recovery environment, and rehabilitation program, resulting in a higher KSS, which may be unrelated to the patients’ nutritional status. Furthermore, the GNRI calculation sets the weight/ideal weight to 1 when the weight exceeds the ideal weight according to its initial design^2^. I did not see the author mention that in this article; if they did not do that, then some bias may be encountered. Furthermore, the exclusion of patients with a body mass index (BMI) >35 kg/m^2^ created some bias in the interpretation of the data.

At last, I found some possible over-conclusions in this article. For instance, the abstract results include the following: First, ‘Our results showed that despite having a lower postoperative HKA angle deviation, the RA-TKA group had a similar postoperative KSS score compared with the conventional TKA group in elderly adults.’ Actually, it is in the elderly adults with GNRI >100; for the elderly adults investigated in this study, there were no differences between HKA and KSS (as in Table 4 of this article). Second, ‘Among elderly patients with GNRI >100, the RA-TKA group achieved significantly more accurate alignment (HKA deviation, *P*=0.039), but did not obtain more advanced postoperative KSS scores because of the compensatory effect of good nutrition status.’ I wonder why good nutrition status would compensate for and cause a lower KSS? Why would good nutrition status enhance the recovery or improvement of KSS? Third, ‘However, among elderly patients with GNRI ≤100, the RA-TKA group had significantly higher postoperative KSS scores compared to the conventional TKA group (*P*=0.025), and this association was not altered after adjustment for other covariates.’ Actually, this association was altered by adjusting the sex, age, preoperative KSS, and BMI, as shown in Model 3 of Table 6 of this article. Furthermore, in the conclusion of the abstract, the author stated that ‘Considering the clinical outcomes of conventional TKA may be more susceptible to the impact of nutrition status, elderly patients with GNRI ≤100 seem to be an applicable population for RA-TKA, which is more stable and would gain significantly more clinical benefits compared with conventional TKA.’ Actually, for those patients with GNRI >100, the improvement regarding HKA, coronal femoral component angle (CFCA), and sagittal tibial component angle (STCA) is more obvious (as shown in Table 8 of this article). Why do the authors only propose the benefit to those elderly patients with GNRI I≤100 but not for those elderly patients with GNRI >100?

Overall, it seems that in the approach to demonstrating that RA-TKA is superior to TKA, nutritional factors were added on. However, certain explanations lack sufficient data to support this claim, and there is a possibility of drawing unwarranted conclusions.

## Ethical approval

Not applicable.

## Consent

Not applicable.

## Sources of funding

The funding for this research was provided by the Chang Gung Memorial Hospital through grant number CORPG8M0331, awarded to Ching-Hua Hsieh.

## Author contribution

C.-Y.H.: manuscript writing; C.-H.H.: study concept.

## Conflicts of interest disclosure

The authors declare no conflicts of interest.

## Research registration unique identifying number (UIN)

Not applicable.

## Guarantor

Both authors.

## Data availability statement

Not applicable.

## Provenance and peer review

Not commissioned, externally peer-reviewed.
